# Emerging cardiovascular toxicity associated with CDK4/6 inhibitors: real-world insights from the FDA adverse event reporting system

**DOI:** 10.3389/fphar.2025.1558128

**Published:** 2025-05-30

**Authors:** Wensheng Liu, Feifei Gao, Xue Song, Hao Chen, Youjun She, Jiyong Liu, Qiong Du

**Affiliations:** ^1^ Department of Pharmacy, Fudan University Shanghai Cancer Center, Shanghai, China; ^2^ Department of Oncology, Shanghai Medical College, Fudan University, Shanghai, China; ^3^ Clinical Research Center, Renji Hospital, Shanghai Jiao Tong University School of Medicine, Shanghai, China

**Keywords:** CDK4/6 inhibitors, cardiovascular adverse events, pharmacovigilance, disproportionality analysis, FAERS

## Abstract

**Background:**

Despite the unprecedented advancement of cyclin-dependent kinase 4 and 6 inhibitors (CDK4/6i) in the treatment paradigm for hormone-dependent breast cancer, reports of cardiovascular adverse events (CVAEs) in both pivotal trials and real-world settings have garnered concerns.

**Objectives:**

we aim to profile the incidence, clinical characteristics and risk factors of CVAEs associated CDK4/6i to provide a vigilant reference for cancer management.

**Methods:**

The global disproportionality study was conducted by utilizing safety reports submitted to the FDA adverse event reporting system (FAERS) during the period from January 2015 to September 2024. Reporting odds ratio (ROR) was employed to identify and evaluate emerging CVAEs related to CDK4/6i. Multivariable logistic regression analysis was utilized to explore factors associated with CVAEs following CDK4/6i treatment. Parametric and cumulative distribution was used for the reported time-to-onset analysis.

**Results:**

A total of 4,709 reports of CVAEs were identified with CDK4/6i, of which 4264 (90.5%) were classified as serious and 12.0% were fatal situation. The median onset time of CVAEs with CDK4/6i was 102 days (interquartile range [IQR], 25-374 days). Disproportionality analysis revealed that Abemaciclib was significantly increased signal of venous thromboembolism (ROR = 2.57 [2.24-2.96]), whereas cardiac arrhythmia (ROR = 2.51 [2.13-2.96]) and torsade de pointes/QT prolongation (ROR = 5.7 [5-6.5]) were showed significantly disproportionate for ribociclib. Meanwhile, cerebrovascular accident and thrombosis were showed significant associated with Abemaciclib ribociclib or palbociclib treatment. Some emerging potential CVAEs, such as myocardial infarction and pulmonary edema, were found to be significantly associated with ribociclib and palbociclib. Additionally, age exceeding 65 years and types of CDK4/6i were significant risk factors for the incidence of CDK4/6i-related CVAEs.

**Conclusion:**

CVAEs might occur with a greater frequency in the context of CDK4/6i than had been previously acknowledged. Our study provide an overview of the incidence, characteristics and risk factors of CDK4/6i-related CVAEs, and also uncovered potential CVAEs that were not identified in the clinical trials.

## 1 Introduction

Breast cancer remains the most frequently diagnosed cancer in women worldwide and the leading cause of cancer-related death, with a significant proportion being of the human epidermal growth factor receptor 2 (HER2)-negative, hormone receptor (HR)-positive subtype (Siegel et al., 2023; Matutino et al., 2018). Endocrine therapy (ET) is the mainstay of systemic therapy for HR + breast cancer, but unfortunately can have limited efficacy due to primary or acquired resistance (Waks and Winer, 2019; Hanker et al., 2020). In recent years, the therapeutic landscape for HR + breast cancer has unprecedentedly evolved by the introduction of cyclin-dependent kinase 4 and 6 inhibitors (CDK4/6i) (Goyal et al., 2023), which can improve the prognosis by combating resistance to traditional ET alone and emerged as a cornerstone in the treatment of advanced or metastatic disease (Morrison et al., 2024). To date, there CDK4/6i, represented by abemaciclib, palbociclib, and ribociclib, have demonstrated significant survival benefits when combined with aromatase inhibitor or fulvestrant, and have been licensed by the United States Food and Drug Administration (FDA) (Hortobagyi et al., 2022; Slamon DJ. et al., 2024; Goetz et al., 2024).

Previous clinical trials have shown that the three available CDK4/6i have similar clinical efficacy, specific safety and tolerability differences then become decisive for physicians’ treatment choices (Onesti and Jerusalem, 2021). Generally, CDK4/6i have a relatively favorable toxicity profile. Among the adverse events (AEs), myelosuppression as well as gastrointestinal and hepatic toxicities are the ones that have been most prevalently documented in clinical trials (Hortobagyi et al., 2022; Slamon DJ. et al., 2024; Goetz et al., 2024). Cardiovascular adverse events (CVAEs) are considered to be rare events associated with CDK4/6i therapy, but they can be serious and even disruptive to the treatment schedule and detrimental to the prognosis (Fradley et al., 2023; Liu et al., 2023). A recent pooled analysis of the MONARCH 2 and 3 trials showed that venous thromboembolic events (VTEs) occurred in 4.8% and 6.1% of patients treated with abemaciclib plus fulvestrant and abemaciclib plus aromatase inhibitor, respectively (Rugo et al., 2021). Additionally, the results of the MONALEESA-2 safety analysis, in which 3.3% of patients experienced prolonged QT intervals (>480 ms) after treatment with ribociclib and letrozole, have further highlighted the importance of routine cardiac monitoring (Hortobagyi et al., 2022; Prat et al., 2024). Of note, QTc prolongation was only observed with ribociclib and has not been shown to be a class-related effect, but cardiac events continue to be reported in post-marketing clinical practice for both palbociclib and abemaciclib (Fiste et al., 2024; Oyakawa et al., 2021; Cicini et al., 2022).

The heterogeneity between clinical trial populations and real-world patients, along with the extended durations of drug exposure in everyday practice, which may not fully capture the breadth of CVAEs associated with CDK4/6i (Fradley et al., 2023; Fiste et al., 2024; Oyakawa et al., 2021; Cicini et al., 2022; Papageorgiou et al., 2021). A recent large-sample retrospective study based on the OneFlorida database found that 24% of CDK4/6i-treated patients experienced CVAEs, which was even slightly higher than in the anthracycline-treated group (p = 0.063) (Fradley et al., 2023). This underscores the critical need for large sample size, real-world data to accurately assess the incidence, onset, and management of CVAEs associated with CDK4/6i. The FDA Adverse Event Reporting System (FAERS) represents a worldwide, highly authoritative, and open-source database dedicated to the spontaneous reporting of adverse events (AEs). It encompasses potential AEs that have manifested in extensive real-world samples of drugs, under circumstances not covered by controlled studies (Liu et al., 2024).

In light of the above considerations, the present study aims to elaborate the incidence, characteristics, risk factors and outcomes of CDK4/6i-related CVAEs leveraging the FAERS database and to unveil potential CVAEs risk ignored in the clinical trials and thereby provide a vigilant reference for therapeutic decision-making and optimize patient management.

## 2 Materials and methods

### 2.1 Data source

This observational, retrospective, pharmacovigilance study was based on AEs reported in the FAERS database, a publicly accessible post-marketing safety surveillance database which contains data on patient gender, age, indication, regimen, AEs, therapy start date, event date, etc. (https://fis.fda.gov/extensions/FPD-QDE-FAERS/FPD-QDE-FAERS.html). Individual case safety reports submitted between January 2015 (the period when first CDK4/6i product palbociclib (IBRANCE^®^; Pfizer Inc.) officially approved by the FDA) and September 2024 were sourced to constitute our original study dataset. To adhere to the FDA guidelines, duplicate reports (i.e., the reports overlap in terms of drug, age, sex, date of event, reporting country, indication and AE) from the same patient were excluded, and only the most recent report was included. As the data were made anonymous, the approval of an ethics committee (i.e., IRB approval) was not necessary for the present study.

### 2.2 Identification of CDK4/6i reports with CVAEs

The study population was defined as reports where the drug indication was breast cancer and only cases where CDK4/6i was the primary suspect (PS) drug were included for exposure assessment. The target drug is identified by matching generic names (abemaciclib, palbociclib, and ribociclib) and brand names (Verzenios^®^, IBRANCE^®^, Kisqali^®^) to the “prod_ai” and “drugname” fields in the DRUG table. To summarize and analyze specific CVAEs and patients regarding CDK4/6i in a structured way, all CVAEs and indications in our collection were coded using the PTs and then mapped to their corresponding Standardized MedDRA Queries (SMQs) levels in MedDRA (version 25.0). The breast cancer population in our study was defined by SMQ (SMQ code = 20000149) using the search field “indi_pt”, and the particular preferred terms (PTs) included are shown in Supplementary Table S1. Cases were identified as reports with at least one of the following CVAEs as retrieved using the following SMQs—cardiac arrhythmia (SMQ code = 20000049), cardiac failure (SMQ code = 20000004), cardiomyopathy (SMQ code = 20000150), embolic and thrombotic events, arterial (SMQ code = 20000082), embolic and thrombotic events, vessel type unspecified and mixed arterial and venous (SMQ code = 20000083), embolic and thrombotic events, venous (SMQ code = 20000084), hypertension (SMQ code = 20000147), myocardial infarction (SMQ code = 20000047), noninfectious myocarditis/pericarditis (SMQ code = 20000239), other ischaemic heart disease (SMQ code = 20000168), pulmonary hypertension (SMQ code = 20000130), and torsade de pointes/QT prolongation (SMQ code = 20000001). Furthermore, searches were conducted only using a narrow terms of SMQs to improve specificity and increase the positive predictive value for case identification (Géniaux et al., 2014). Detailed PTs regarding to CVAEs are available in Supplementary Table S2.

### 2.3 Pharmacovigilance signal detection

Disproportionality analyses are primarily employed in pharmacovigilance studies to assess possible associations between specific AEs and drugs, and to further investigate clinical associations in individual cases (Caster et al., 2020). The reporting odds ratio (ROR) methodology was employed to identify signals and conduct a characteristic analysis, thereby evaluating the correlation between overreported CVAEs and CDK4/6i treatment regimens. An two-by-two contingency table was created as a basis for ROR calculation (Supplementary Table S3), where “a” denotes the number of cases with CVAEs of CDK4/6i alone; “b” denotes the number of cases with other AEs of CDK4/6i; “c” denotes the number of cases with CVAEs of other drugs except for CDK4/6i; “d” denotes the number of cases with other AEs of other drugs except for CDK4/6i. The calculation of the ROR value and its 95% confidence interval (CI) was as follows:

$$\text{ROR}=\frac{\left(a/c\right)}{\left(b/d\right)}=\frac{\text{ad}}{\text{bc}}$$

$$\text{ROR}025=e^{\ln\left(\text{ROR}\right)-1.96\sqrt{\;\left(\frac{1}{a}+\frac{1}{b}+\frac{1}{c}+\frac{1}{d}\right)\;}}$$

$$\text{ROR}075=e^{\ln\left(\text{ROR}\right)+1.96\sqrt{\;\left(\frac{1}{a}+\frac{1}{b}+\frac{1}{c}+\frac{1}{d}\right)\;}}$$

A positive pharmacovigilance signal was considered significant when the number of suspected CVAE was three or more, with the ROR025 (lower limit of the 95% CI for the ROR) was greater than one (Liu et al., 2024). Overall, cardiovascular PTs meeting the above criteria were defined as CDK4/6i-related CVAEs and retained for further analysis (N = 4,709).

### 2.4 Cumulative incidence and time-to-onset analysis

The time-to-onset of CDK4/6i-related CVAEs is defined as the time interval between the CVAE onset date in the DEMO file (EVENT_DT) and the date of CDK4/6i initiation in the THER file (START_DT). The Kaplan-Meier method was used to plot the cumulative incidence of diverse CVAEs related to different CDK4/6i. The Weibull shape parameter test, which describes the probability of an AE increasing or decreasing over time, was used to statistically analyze time to onset of CVAEs related to CDK4/6i (Kinoshita et al., 2020). The parametric distribution is represented by scale parameter α and shape parameter β. For the purposes of this study, the shape parameter β of the Weibull distribution was used for the representation of the hazard in the absence of a reference population. The risk of AEs is thought to decrease over time if the shape parameter β is less than 1 and its 95% CI is less than 1 (early failure type curve); it is thought to be constant over time if the shape parameter β is roughly equal to or near 1 and its 95% CI includes the value 1 (random failure type curve); and it is thought to increase over time if the shape parameter β > 1 and its 95% CI excludes the value 1 (wear failure type curve) (Kinoshita et al., 2020; Yang et al., 2024).

### 2.5 Statistical analysis

Descriptive analysis was utilized to encapsulate the demographic profiles and characteristics of cases experiencing CDK4/6i-related CVAEs. A multivariate logistic regression analysis was employed to explore the incidence of CDK4/6i-related CVAEs across a spectrum of risk factors, including age, weight, and medication regimens. The impact of each predictor was quantified using odds ratios (ORs) and their corresponding 95% CIs, while statistical significance was assessed through parameter estimates, standard errors (Std. Error), z-values, and p-values. The log-rank test served to compare the median onset times of CVAEs among patients receiving different CDK4/6i treatments. Statistical significance was defined as a p-value below 0.05. All data management, statistical analysis and graphical representations in this study were executed using R software, version 4.3.3.

## 3 Results

### 3.1 The current overview of CVAEs profile following CDK4/6i therapy

**FIGURE 1:**
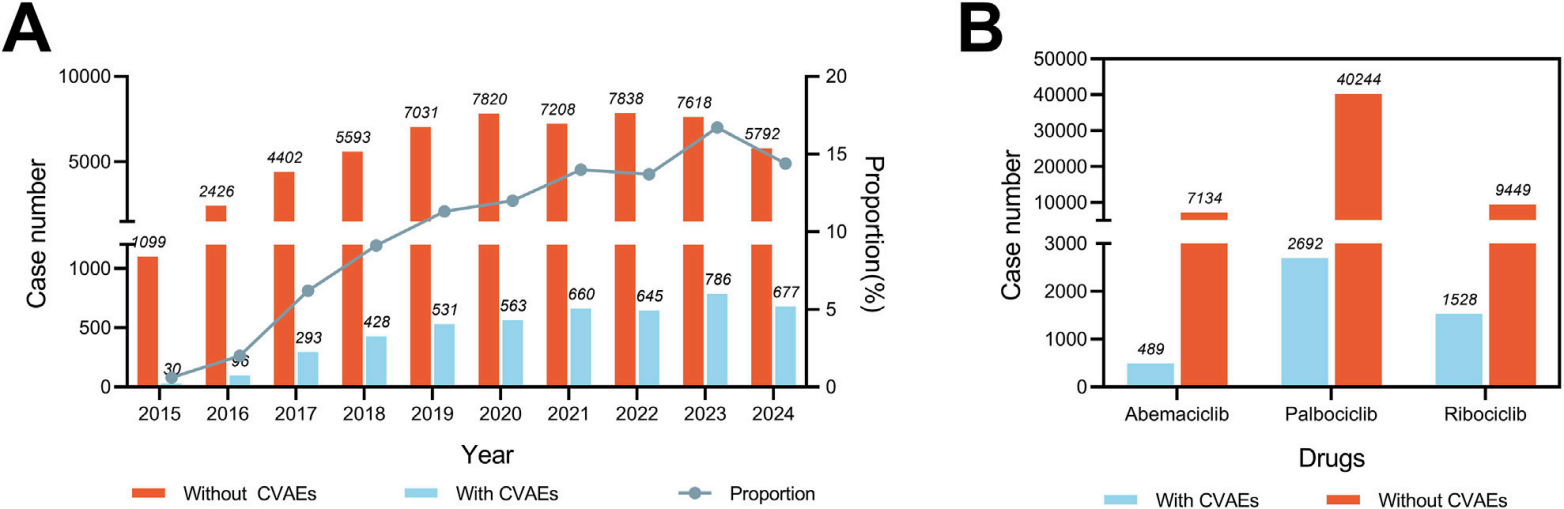
Statistics of CDK4/6i reports with and without CVAEs in the FAERS from 2015 to 2024Q3. **(A)** Bar chart showing the number of CDK4/6i reports with and without CVAE per year, and a proportional line chart showing the proportion of CDK4/6i reports with and without CVAE per year **(B)** Bar chart showing the number of reports of CVAE with and without CVAE for different CDK4/6i.

From the FAERS database, 61,536 reports from breast cancer patients identified CDK4/6i as their PS drug spanning from January 2015 to September 2024, with 4,709 (7.65%) of these reports detailing CVAEs. As depicted in Figure 1A, the proportion of CDK4/6i reports with CVAEs among all CDK4/6i reports has exhibited an upward trend, rising from 2.73% (30 out of 1,099) in 2015 to 11.69% (677 out of 5,792) in 2024. Additionally, the proportion of CVAEs reported for each CDK4/6i, ribociclib had the highest rate (16.17%), followed by abemaciclib (6.85%) and palbociclib (6.69%), highlighting the differences in the frequency of reported CVAEs for these CDK4/6i (Figure 1B).

### 3.2 Descriptive analysis of reports with CDK4/6i-related CVAEs

**FIGURE 2:**
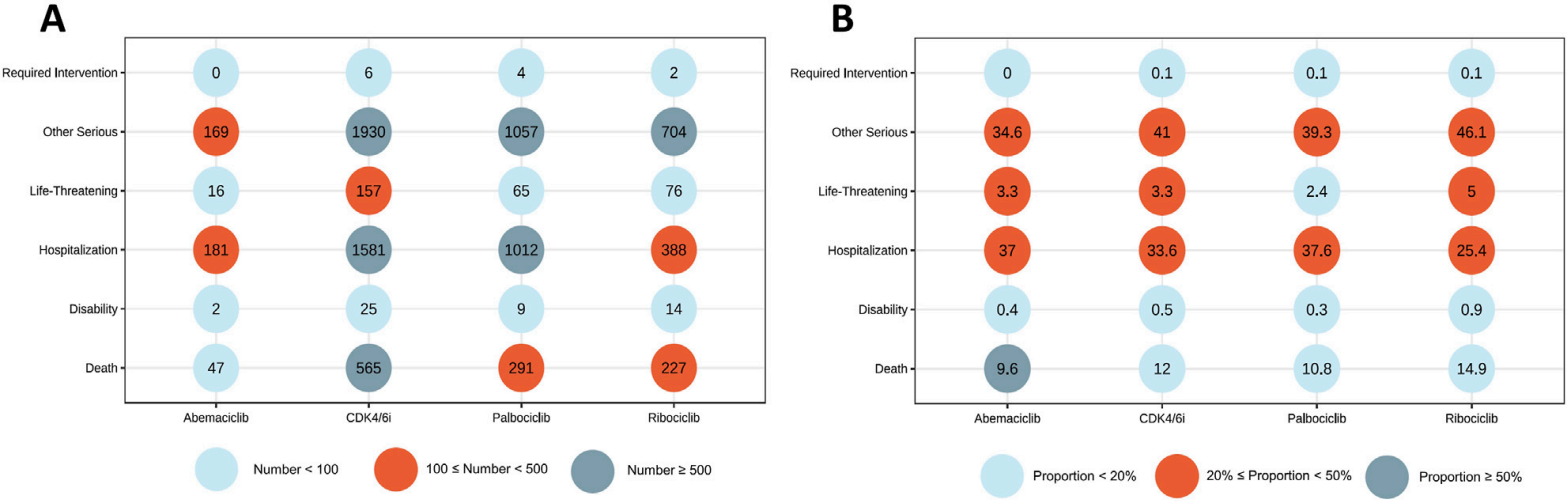
Outcomes of cases with CDK4/6i-related CVAEs. **(A)** Number of serious outcomes observed in CDK4/6i-related CVAE cases. **(B)** Percentage of serious outcomes observed in CDK4/6i-related CVAE cases.

The demographic features of patients experiencing CDK4/6i-related CVAEs are detailed in Table 1. Out of 4,607 reports that specified the gender of the reporters, an overwhelming majority of 4,551 (96.64%) were submitted by females. The median age was 67 years (IQR 58–75). The median weight was 71 kg (IQR: 61–83). In terms of geographical distribution, the United States led with the most reports at 42.32%, followed by India (7.90%), Argentina (6.69%), Germany (5.67%), and France (3.70%). Figures 2A, B depict the number and percentage of serious outcomes associated with different CDK4/6i, respectively. Among the 4,264 cases with reported outcomes, the rates of mortality, disabilities, and hospitalizations were 12.0%, 0.5%, and 33.6%, respectively.

**TABLE 1:** Demographic and clinical information of reports with CDK4/6i-related cardiovascular adverse events sourced from the FAERS database (From 1 January 2015 to 30 September 2024).

	Abemaciclib	Palbociclib	Ribociclib	CDK4/6i
Number of CVAEs (n)	N = 489	N = 2,692	N = 1,528	N = 4709
Gender [n, (%)]				
Female	453 (92.64)	2,638 (97.99)	1,460 (95.55)	4551 (96.64)
Male	9 (1.84)	34 (1.26)	13 (0.85)	56 (1.19)
Missing	27 (5.52)	20 (0.74)	55 (3.60)	102 (2.17)
Weight [n, (%)]				
<50 kg	25 (5.11)	54 (2.01)	35 (2.29)	114 (2.42)
50-100 kg	87 (17.79)	869 (32.28)	448 (29.32)	1,404 (29.82)
>100 kg	3 (0.61)	89 (3.31)	31 (2.03)	123 (2.61)
Median (IQR)	60 (50,72)	73 (62,85)	70 (61,80)	71 (61,83)
Age, year [n, (%)]				
<18	0 (0)	0 (0)	0 (0)	0 (0)
18-64.9	135 (27.61)	905 (33.62)	517 (33.84)	1,557 (33.06)
65-85	144 (29.45)	1,515 (56.28)	434 (28.40)	2093 (44.45)
>85	6 (1.23)	97 (3.60)	16 (1.05)	119 (2.53)
Median (IQR)	65 (56,73)	69 (61,76)	64 (53,72)	67 (58,75)
Reporter [n, (%)]				
Consumer	296 (60.53)	1,298 (48.22)	751 (49.15)	2,345 (49.80)
Healthcare professional	183 (37.42)	1,348 (50.07)	766 (50.13)	2,297 (48.78)
Missing	10 (2.04)	46 (1.71)	11 (0.72)	67 (1.42)
Reporting Country (Top five) [n, (%)]				
	USA 209 (42.74)	USA 1610 (59.81)	Germany 211 (13.81)	USA 1993 (42.32)
	Japan 67 (13.70)	India 282 (10.48)	USA 174 (11.39)	India 372 (7.90)
	China 36 (7.36)	Argentina 258 (9.58)	Brazil 85 (5.56)	Argentina 315 (6.69)
	Italy 24 (4.91)	France 110 (4.09)	India 83 (5.43)	Germany 267 (5.67)
	France 16 (3.27)	Canada 78 (2.90)	UK 62 (4.06)	France 174 (3.70)

Note: FAERS, FDA adverse event reporting system; IQR, interquartile range.

### 3.3 Scanning for CDK4/6i-related CVAEs

As detailed in Supplementary Table S4, the prevalent CVAEs reported subsequent to CDK4/6i treatment were thrombosis, occurring in 567 cases (10.30%), hypertension in 523 cases (9.50%), and cerebrovascular accidents in 480 cases (8.72%). Within the abemaciclib-related reports, pulmonary embolism emerged as the most frequently documented CVAE, affecting 90 individuals (15.60%). For palbociclib, thrombosis was identified as the leading CVAE, with 366 cases (11.94%). In the case of ribociclib, the most commonly reported CVAE was electrocardiogram QT prolonged, which was observed in 305 cases (16.40%).

**FIGURE 3:**
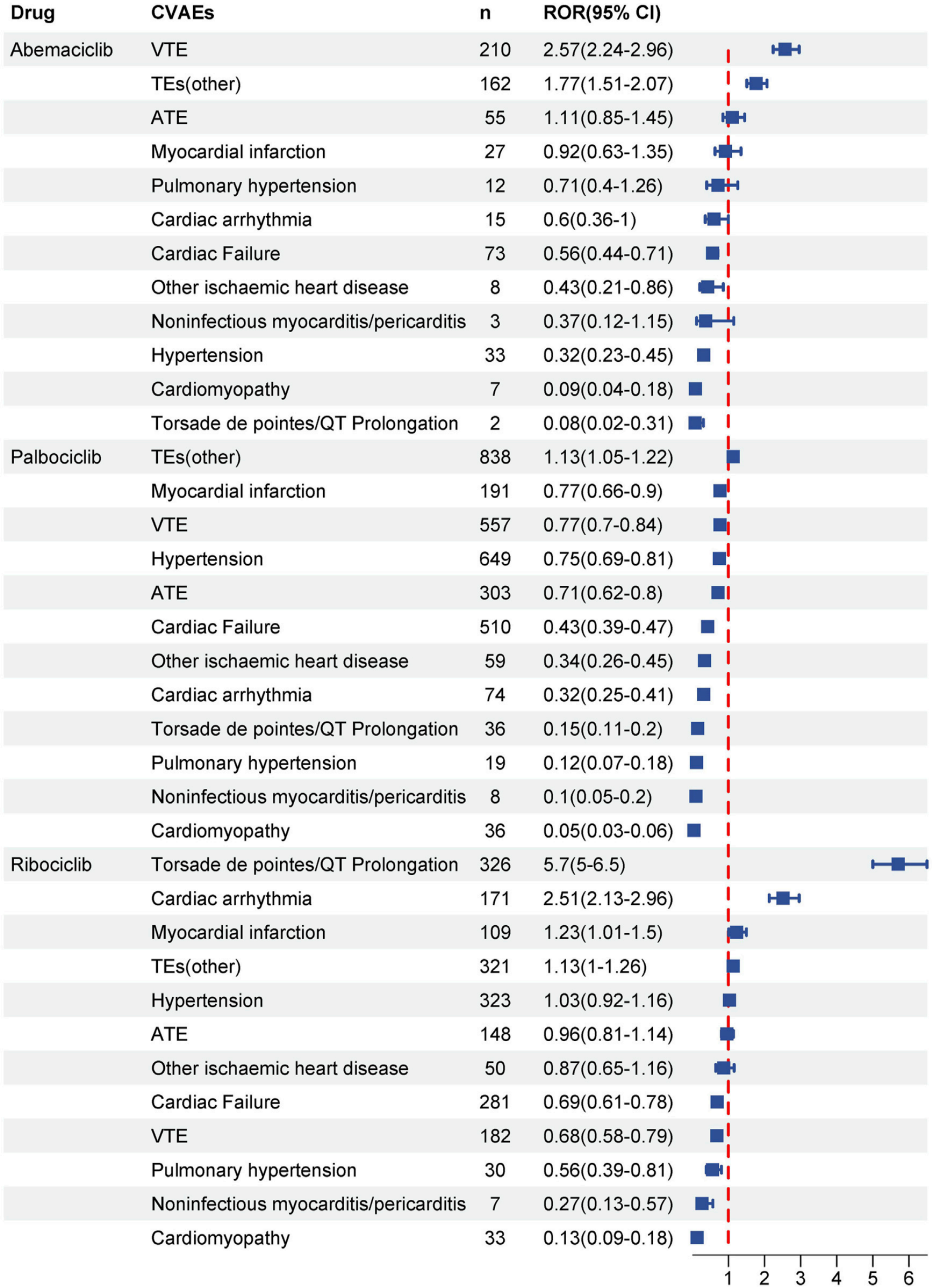
Disproportionality analysis of significant CVAEs associated with CDK4/6i at the standardized MedDRA Query (SMQ) level. Signals at the SMQ level indicate the strength and significance of the association between medication and specific cardiovascular outcome. Significant signals were detected when the lower limit of the reporting odds ratio (ROR) was greater than 1 and the number of cases was greater than or equal to three.

As illustrated in Figure 3, systematic SMQ level evaluations identified significant signals associated with ribociclib across various cardiovascular categories, notably in cardiac arrhythmia (n = 171, ROR025 = 2.13), myocardial infarction (n = 109, ROR025 = 1.01), and torsade de pointes/QT prolongation (n = 326, ROR025 = 5). In contrast, neither abemaciclib nor palbociclib demonstrated positive signals within these specific CVAEs. Abemaciclib, however, exhibited prominent signal strengths in embolic and thrombotic events, venous (n = 210, ROR025 = 2.24) and embolic and thrombotic events, vessel type unspecified and mixed arterial and venous (n = 162, ROR025 = 1.51). Embolic and thrombotic events, vessel type unspecified and mixed arterial and venous was the most frequently reported CVAEs for palbociclib (n = 838, ROR025 = 1.05). Other SMQs related to CVAEs did not satisfy the pre-established criteria for signal detection.

**FIGURE 4:**
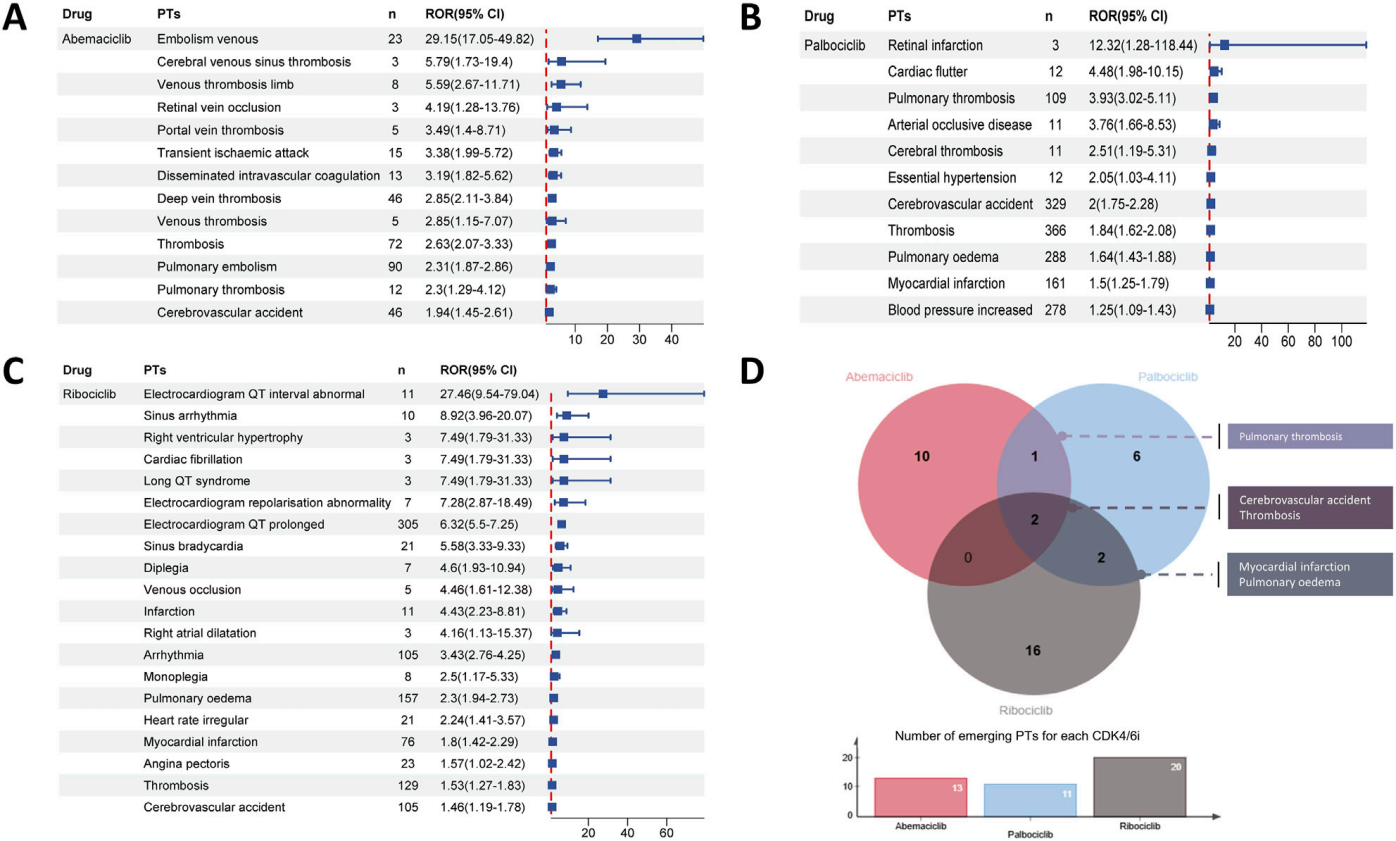
Disproportionality analysis of significant CVAEs associated with CDK4/6i at the Preferred Term (PT) level. Forest plot shows the reporting odds ratio (ROR) values of abemaciclib **(A)** palbociclib **(B)** and ribociclib **(C)** related CVAEs, providing insights into the relative reporting frequency of specific CVAEs beyond expected levels within the dataset. **(D)** The upper venn plots and the lower bar charts indicate the number of duplicated significant CVAEs between different CDK4/6i and the number of disproportionate CVAEs for CDK4/6i at the PT level, respectively.

At the PT level, there were differences in safety signals among three CDK4/6i in respect to CVAEs, with 13 potential CVAEs detected for abemaciclib, 11 for palbociclib and 20 for ribociclib (Figures 4A–C). Among the emerging CVAEs associated with CDK4/6i, CVAEs related to abemaciclib are primarily thrombotic events such as embolism venous, disseminated intravascular coagulation and retinal vein occlusion; while ribociclib-related CVAEs are mainly cardiac events like myocardial infarction, angina pectoris, electrocardiogram QT interval abnormal and cardiac fibrillation, etc.,; and the CVAEs related to palbociclib include both hypertension and diverse thrombotic events as well as cardiac events like pulmonary oedema, myocardial infarction and cardiac fluttter, detailed significant CVAES at the PT level were shown in Figures 4A–C. Furthermore, as indicated in Figure 4D, significant signals for pulmonary thrombosis were detected for both abemaciclib and palbociclib, and significant signals for myocardial infarction and pulmonary oedema were detected for both palbociclib and ribociclib. Notably, cerebrovascular accidents and thrombosis are emerging CVAEs that occurred with all three CDK4/6i.

### 3.4 Analysis of factors influencing CDK4/6i-related CVAEs

A multivariable logistic regression model was applied to assess the various influence factors on CDK4/6i-related CVAEs in breast cancer patients, with considerations given to age, body weight, and the specific type of CDK4/6i (Table 2). Key results indicated that patients aged 65 and older had a significantly higher likelihood of experiencing CVAEs, with an OR of 1.21 (95% CI: 1.16-1.27, p < 0.001). Additionally, body weight did not emerge as a significant predictor for CDK4/6i-related CVAEs (p > 0.05). In terms of the type of CDK4/6i, ribociclib was found to be associated with a significantly higher rate of cardiovascular reports compared to both abemaciclib (OR = 3.20, 95% CI: 2.91-3.53, p < 0.001) and palbociclib (OR = 3.20 vs OR = 1.27).

**TABLE 2:** Multivariable logistic regression analysis of the odds ratio for CDK4/6i-related cardiovascular adverse events controlling for demographic factors.

Predictor variables	Estimate	Std. Error	z value	Or (95%CI)	*p*-Value
Intercept	−2.61	0.06	−44.43	—	—
Age (≥65)	0.19	0.02	8.96	1.21 (1.16–1.27)	*p* < 0.001
Weight (50-100 kg)	0.04	0.04	1.06	1.05 (0.96–1.14)	*p* = 0.288
Weight (>100 kg)	−0.08	0.05	−1.53	0.92 (0.83–1.02)	*p* = 0.125
Palbociclib	0.24	0.05	5.26	1.27 (1.16–1.39)	*p* < 0.001
Ribociclib	1.16	0.05	23.74	3.20 (2.91–3.53)	*p* < 0.001

Note: The analysis adjusts for age (reference: <65 years), weight (reference: <50 kg), CDK4/6i type (reference, abemaciclib);—, NA; OR, odds ratio; CI, confidential interval.

### 3.5 Time to onset analysis

A total of 1,598 available cases experienced CDK4/6i-related CVAEs were included in the time-to-onset analysis. As described in Table 3, the CVAEs associated CDK4/6i treatments initiating with a median onset time of 102 days (IQR: 25-374 days). In our time-to-onset analysis, which was based on parametric distribution, the reported median onset times for CVAEs in relation to abemaciclib, palbociclib, and ribociclib were 102 days, 154 days, and 55 days, respectively. Of note, both the shape parameter β and the upper limit of its 95% were less than 1 in the Weibull shape parameter test, indicating that abemaciclib, palbociclib, and ribociclib demonstrate an early failure type and a gradual decline in the likelihood of CVAEs over time.

**TABLE 3:** Weibull shape parameter test for cardiovascular adverse events associated with CDK4/6i.

Drugs	Case reports	Time to onset (days)		Scale parameter		Shape parameter		Type
		Median (IQR)	Min - max	α	95% CI	β	95%CI	
Abemaciclib	138	102 (24–223)	1–1,329	148.33	114.94–181.72	0.78	0.68–0.88	Early failure
Palbociclib	804	154 (38–452)	1–2,874	278.70	250.78–306.62	0.73	0.69–0.77	Early failure
Ribociclib	656	55 (16–300)	1–2,229	157.17	135.96–178.38	0.60	0.57–0.64	Early failure
CDK4/6i	1,598	102 (25–374)	1–2,874	212.01	195.42–228.60	0.66	0.64–0.69	Early failure

Note: α, scale parameter; represents the scale of the distribution function as the quantile in which 63.2% of CVAEs, occur; β, shape parameter, could be used to confirm the distribution type: early failure type (β < 1), random failure type (95% CI, of β include 1), and wear-out type (β > 1); CI, confidence interval.

**FIGURE 5:**
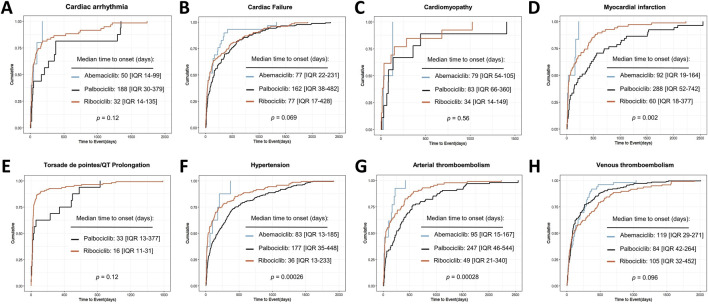
Cumulative distribution curves showing the time to onset of CDK4/6i-related CVAEs in different medication subgroups.

As illustrated in Figure 5, the SMQs of cardiac arrhythmia, cardiac failure, cardiomyopathy, myocardial infarction, torsade de pointes/QT prolongation, hypertension, arterial thrombotic events (ATE) and VTE were selected for time-to-event analysis based on the number of reports for the three CDK4/6i. Based on reports with available time-to-event statistics, the median time-to-event for cardiac arrhythmia was 50 days (IQR 14-99 days) for abemaciclib, 188 days (IQR 30-379 days) for palbociclib, and 32 days (IQR 14-135 days) for ribociclib; For cardiac failure, the median time-to-event was 77 days (IQR 22-23 days) for abemaciclib, 162 days (IQR 38-462 days) for palbociclib, and 77 days (IQR 17-428 days) for ribociclib; For cardiomyopathy, the median time-to-event was 79 days (IQR 54–105 days) for abemaciclib, 83 days (IQR 66-360 days) for palbociclib, and 34 days (IQR 34-149 days) for ribociclib; For myocardial infarction, the median time-to-event was 92 days (IQR 19–164 days) for abemaciclib, 288 days (IQR 52-742 days) for palbociclib, and 60 days (IQR 18-377 days) for ribociclib; For torsade de pointes/QT prolongation, the median time-to-event was 33 days (IQR 13–377 days) for palbociclib, and 16 days (IQR 11-31 days) for ribociclib; For hypertension, the median time-to-event was 83 days (IQR 13–185 days) for abemaciclib,177 days (IQR 35-448 days) for palbociclib, and 36 days (IQR 13-233 days) for ribociclib; For ATE, the median time-to-event was 95 days (IQR 15–167 days) for abemaciclib, 247 days (IQR 46-544 days) for palbociclib, and 49 days (IQR 21-340 days) for ribociclib; For VTE, the median time-to-event was 119 days (IQR 29–271 days) for abemaciclib, 84 days (IQR 42–264 days) for palbociclib, and 105 days (IQR 32–452 days) for ribociclib. Noteworthy, different CDK4/6i treatment strategies may influence the median onset time of CDK4/6i-related myocardial infarction, hypertension, and ATE (p < 0.05) (Figures 5D,F,G).

## 4 Discussion

CDK4/6i have made an unprecedented step forward in treatment paradigm for hormone-dependent breast cancer (Goyal et al., 2023). However, alongside their impressive efficacy, the emergence of cardiac and/or vascular AEs associated with this novel targeted drugs is currently of great interest (Fradley et al., 2023; Liu et al., 2023). While previous reports and studies have documented CVAEs related to CDK4/6i, a significant knowledge gap persists regarding the full spectrum of potential CVAEs linked to this drugs (Yan et al., 2024; Raschi et al., 2021).

Generally, clinical trials have the advantages of rigorous study design, effective control of confounding factors, and confidence in causality. However, the sample size of clinical trials is relatively limited, and the subject population is rigorously screened and not broadly representative, leading to limitations in extrapolating their results to the real world (Hortobagyi et al., 2022; Slamon DJ. et al., 2024; Goetz et al., 2024). The FAERS database, on the other hand, collects data from real-world medical practices, has a large sample size, and covers patients of different ages, genders, races, and disease backgrounds, providing a more comprehensive picture of drug use and AEs in the real world (Liu et al., 2024; Yang et al., 2024). Meanwhile, the continuous updating of the database helps to identify new or rare AEs in a timely manner. Thus, the present study pioneers a comprehensive pharmacovigilance analysis of CVAEs highly relevant to CDK4/6i therapy utilizing real-world data from the FAERS database to explore the clinical characteristics and underlying risk factors associated with such events.

CDK4/6i are generally considered to be safe and well tolerated in the cardiovascular setting, but apart from the known QTc-prolonging effect of ribociclib, data on the prevalence and outcomes of CVAEs associated with these drugs remain scarce (Onesti and Jerusalem, 2021; Yang et al., 2021). Currently, the meta-analysis result of pivotal clinical trials associated with approved CDK4/6i revealed that palbociclib is associated with QTc prolongation, with a relative risk even twice that of ribociclib (Murad et al., 2024). Besides, pooled analysis of MONARCH 2 and 3 trials found that any-grade VTE occurred in 4.8% and 6.1% of patients treated with abemaciclib plus fulvestrant and abemaciclib plus aromatase inhibitor, respectively (Rugo et al., 2021). Regarding post-marketing observational evidence, a retrospective cohort study leveraging the OneFlorida Data Trust database revealed that approximately 24% of the 1,376 patients treated with CDK4/6i encountered CVAEs, which was even slightly higher than in the anthracycline-treated group (p = 0.063) (Fradley et al., 2023). Meanwhile, cases of CDK4/6i-related CVAEs, including atrioventricular block, myocardial dysfunction, and takotsubo cardiomyopathy, have also been reported during the clinical practice (Fiste et al., 2024; Oyakawa et al., 2021; Cicini et al., 2022). Overall, CDK4/6i-induced CVAEs may be more common than previously recognized and the spectrum of symptoms appears to be broader.

As depicted in Figure 1, the quantity of CDK4/6i reports within the FAERS database demonstrated a consistent upward progression from 2015 to 2023. Additionally, the proportion of CDK4/6i reports associated with CVAEs presented an increasing tendency, ascending from 2.66% in 2015 to 10.47% during the period from the first quarter to the third quarter of 2024. This trend may signify a genuine escalation in the incidence of these events, as well as an enhanced awareness of such risks among healthcare providers and patients. Meanwhile, approximately 90.5% of patients (n = 4264) experienced serious outcomes with available follow-up data (Figures 2A,B). Of note, the study showed that ribociclib-associated CVAEs resulted in a higher proportion of deaths compared to other CDK4/6i, although these results are preliminary and warrant further validation in larger-scale or prospective studies. Consequently, as the prevalence of CDK4/6i usage broadens (Slamon D. et al., 2024), the concomitant cardiovascular risks will mandate periodic updates of clinical management guidelines to refine therapeutic strategies and improve the quality of life for patients receiving these treatments.

As illustrated in Figure 3, our disproportionality analysis revealed that the most significant CVAEs for abemaciclib at the SMQ level was VTE (n = 210; ROR025:2.24), which was in line with evidence from RCTs (Rugo et al., 2021) and further strengthens the conclusion that it may increase the risk of VTE in breast cancer patients (Hua et al., 2024; Watson et al., 2022). Furthermore, specific CVAEs at the PT level associated CDK4/6i were first disclosed in the present study. Data from the pivotal PALOMA-2 trial showed that 0.9% of the 444 patients in the pabocinib/letrozole combination trial arm experienced pulmonary embolism (Finn et al., 2016). Similarly, in our pharmacovigilance analysis, pulmonary thrombosis were detected as a significant disproportionality signal for palbociclib at the PT level (n = 109; ROR025:3.02) (Figure 4B). Of note, cerebrovascular accidents and thrombosis are emerging CVAEs that occurred with all three CDK4/6i (Figure 4D), the interpretation of these data is unclear. On the whole, our results support that thrombotic events appear to be a class of risk that exists for CDK4/6i (Raschi et al., 2021; Watson et al., 2023). Despite an extensive clinical evidence base implicating thrombosis in patients treated with CDK 4/6i, no studies have yet elucidated the underlying mechanisms driving this prothrombotic state (Watson et al., 2023). Considering that VTE ranks as the second major cause of death in cancer patients following cancer progression, there is an urgent need for validated risk scores and effective thromboprophylaxis strategies specifically tailored to CDK4/6i-associated VTE (Khorana et al., 2007; West et al., 2021).

In our disproportionality analysis, in addition to the well-recognized torsade de pointes/QT prolongation (Yan et al., 2024; Murad et al., 2024), ribociclib had emerging signals, such as cardiac arrhythmia (n = 171, ROR025 = 2.13) and myocardial infarction (n = 109, ROR025 = 1.01), which both showed significant disproportionality (Figure 3). Besides, some unexpected cardiac safety concerns such as pulmonary oedema, angina pectoris, right ventricular hypertrophy, and right atrial dilatation were also raised for ribociclib in our study (Figure 4C). Although neither abemaciclib nor palbociclib detected a positive signal in cardiotoxicity at the SMQ level, four emerging cardiac events were observed for the first time with palbociclib (Figure 4B), including pulmonary oedema, blood pressure increased, myocardial infarction, and cardiac flutter. Therefore, it’s imperative to continuously monitor and regularly update the drug labeling with emerging cardiovascular risks, enabling clinicians to promptly identify and proactively manage targeted patients.

Furthermore, as shown in Figure 5 and Table 3, ribociclib may induce CVAEs more acutely than abemaciclib and palbociclib (median onset time was 55 days, 102 days and 154 days, respectively). This phenomenon may involve ribociclib-mediated dysregulation of cardiac ion channel genes, specifically upregulation of SCN5A (encoding the NaV1.5 sodium channel) and SNTA1 (syntrophin-α1), coupled with downregulation of KCNH2 (encoding the hERG potassium channel critical for cardiac repolarization) (Santoni et al., 2019). Although the exact molecular mechanism by which CDK4/6i induce CVAEs has not yet been fully elucidated, the recent basic and translational studies have suggested that CDK4/6i may contribute to CVAEs through the modulation of potassium and sodium channel activity, exacerbation of vascular inflammation, promotion of left ventricular remodeling, and the downregulation of the PI3K/AKT signaling pathways (Papageorgiou et al., 2021; Abu-Khalaf et al., 2023). Furthermore, the occurrence of CVAEs is associated with multiple factors, such as age, basic cardiovascular and metabolic conditions, and medications, etc. (Wilcox et al., 2024) In terms the factors of age, aging impacts the activity of CDK4/6i by disrupting cell cycle regulation and is compounded by its association with patient comorbidities, consequently elevating the risk of CVAEs linked to cancer treatment (Battisti et al., 2018). Our study findings indicate that individuals aged 65 and older have a significantly higher incidence of reported CVAEs (Table 2), underscoring the importance of considering age-specific efficacy and tolerability of CDK4/6i and highlighting the need for vigilant cardiovascular surveillance in this age group.

Taken together, CVAEs related to CDK4/6i seems more common than previously recognized. The results of our study suggested that cardiologists, oncologists, and pharmacists should pay special attention to the possible CVAEs of CDK4/6i, since which can be serious. Meanwhile we suggested that the need to develop class-specific cardiac and/or vascular toxicity management and mitigation strategies for CDK4/6i treatment, especially in patients with high risk factors. The advent of multi-omics technologies (e.g., genomics, epigenomics, transcriptomics, proteomics, and metabolomics) has demonstrated potential to identify and validate candidate biomarkers for CVAEs, enabling enhanced characterization of underlying pathophysiological mechanisms and accelerating the discovery of cardioprotective therapeutics (Rhee et al., 2020). A more comprehensive and accurate understanding of the real-world profile of CVAEs related to CDK4/6i is conducive to optimizing the medication regimen of CDK4/6i and enhancing the quality of life for breast cancer patients.

The present study, like other pharmacovigilance studies based on spontaneous reporting systems, acknowledged certain limitations. First, the FAERS database is inherently constrained by issues of underreporting, incompleteness, and selectivity in reporting. Adverse events that are less severe or more common might be underreported, whereas those that are more serious or rare could be overreported. Second, confounding effects related to disease severity, prior chemotherapy exposure, preexisting cardiovascular conditions, and baseline patient characteristics might limit detecting CVAEs in a standard comparison. For example, tamoxifen carries a risk of thrombosis, acute myocardial infarction, and ischemic stroke. Concomitant use of tamoxifen and CDK4/6i may increase the incidence of CVAEs (Zhang et al., 2025). Third, the nature of spontaneous reporting systems means that they do not include data on the total number of patients treated with CDK4/6i, precluding the calculation of precise incidence rates for CVAEs. The cardiovascular risks for CDK4/6i discussed thus reflect only the proportion of these events reported among all AEs, not their true incidence in the broader patient population. Finally, disproportionality analysis serves as a hypothesis-generation process and merely indicates potential statistical correlations, devoid of establishing causality. Hence, it is crucial to underscore that definitive determination of causality mandates further in-depth research and rigorous verification (Almenoff et al., 2007). Despite the results of present study should be considered as indicative only, it can provide a vigilant evidence for clinicians to identify intervention early in possible CVAEs associated with CDK4/6i treatment.

## 5 Conclusion

Our study provide an overview of the incidence, characteristics and risk factors of CDK4/6i-related CVAEs, and also uncovered potential CVAEs that were not identified in the clinical trials. Through comprehensive analysis of the FAERS database, CVAEs related to CDK4/6i seems more common than previously recognized. Additionally, CDK4/6i has been systematically identified for the first time with cardiac and/or vascular signals at the SMQ and PT level. Age exceeding 65 years was a significant risk factor for the incidence of CDK4/6i-related CVAEs. Given the growing importance of cardio-oncology, the findings call for heightened awareness and proactive management of potential cardiovascular risks associated with CDK4/6i, particularly in specific high-risk populations. Further prospective epidemiological and experimental studies are warranted to validate any causative relationship and to explore the underlying pathophysiology of this novel medications, respectively.

## Data Availability

The original contributions presented in the study are included in the article/Supplementary Material, further inquiries can be directed to the corresponding authors.

## References

[B1] Abu-Khalaf M. M. Alex Hodge K. Hatzis C. Baldelli E. El Gazzah E. Valdes F. (2023). AKT/mTOR signaling modulates resistance to endocrine therapy and CDK4/6 inhibition in metastatic breast cancers. NPJ Precis. Oncol. 7 (1), 18. 10.1038/s41698-023-00360-5 36797347 PMC9935518

[B2] Almenoff J. S. Pattishall E. N. Gibbs T. G. DuMouchel W. Evans S. J. Yuen N. (2007). Novel statistical tools for monitoring the safety of marketed drugs. Clin. Pharmacol. Ther. 82 (2), 157–166. 10.1038/sj.clpt.6100258 17538548

[B3] Battisti N. M. L. De Glas N. Sedrak M. S. Loh K. P. Liposits G. Soto-Perez-de-Celis E. (2018). Use of cyclin-dependent kinase 4/6 (CDK4/6) inhibitors in older patients with ER-positive HER2-negative breast cancer: young International Society of Geriatric Oncology review paper. Ther. Adv. Med. Oncol. 10, 1758835918809610. 10.1177/1758835918809610 30479671 PMC6249663

[B4] Caster O. Aoki Y. Gattepaille L. M. Grundmark B. (2020). Disproportionality analysis for pharmacovigilance signal detection in small databases or subsets: recommendations for limiting false-positive associations. Drug Saf. 43 (5), 479–487. 10.1007/s40264-020-00911-w 32008183 PMC7165139

[B5] Cicini M. P. Ferretti G. Morace N. Nisticò C. Cognetti F. Rulli F. (2022). Second-degree type 2 atrioventricular block requiring permanent cardiac pacing in patients on CDK4/6 inhibitors: report of two cases. Breast Care (Basel) 17 (3), 330–335. 10.1159/000519728 35957944 PMC9247538

[B6] Finn R. S. Martin M. Rugo H. S. Jones S. Im S. A. Gelmon K. (2016). Palbociclib and letrozole in advanced breast cancer. N. Engl. J. Med. 375 (20), 1925–1936. 10.1056/NEJMoa1607303 27959613

[B7] Fiste O. Trika C. Syrigos N. K. Kotteas E. A. (2024). A rare case of Takotsubo cardiomyopathy. Eur. Rev. Med. Pharmacol. Sci. 28 (5), 2063–2067. 10.26355/eurrev_202403_35619 38497887

[B8] Fradley M. G. Nguyen N. H. K. Madnick D. Chen Y. DeMichele A. Makhlin I. (2023). Adverse cardiovascular events associated with cyclin-dependent kinase 4/6 inhibitors in patients with metastatic breast cancer. J. Am. Heart Assoc. 12 (12), e029361. 10.1161/JAHA.123.029361 37301767 PMC10356048

[B9] Géniaux H. Assaf D. Miremont-Salamé G. Raspaud B. Gouverneur A. Robinson P. (2014). Performance of the standardised MedDRA® queries for case retrieval in the French spontaneous reporting database. Drug Saf. 37 (7), 537–542. 10.1007/s40264-014-0187-2 24942754

[B10] Goetz M. P. Toi M. Huober J. Sohn J. Trédan O. Park I. H. (2024). Abemaciclib plus a nonsteroidal aromatase inhibitor as initial therapy for HR+, HER2-advanced breast cancer: final overall survival results of MONARCH 3. Ann. Oncol. 35 (8), 718–727. 10.1016/j.annonc.2024.04.013 38729566

[B11] Goyal R. K. Chen H. Abughosh S. M. Holmes H. M. Candrilli S. D. Johnson M. L. (2023). Overall survival associated with CDK4/6 inhibitors in patients with HR+/HER2-metastatic breast cancer in the United States: a SEER-Medicare population-based study. Cancer 129 (7), 1051–1063. 10.1002/cncr.34675 36760031

[B12] Hanker A. B. Sudhan D. R. Arteaga C. L. (2020). Overcoming endocrine resistance in breast cancer. Cancer Cell 37 (4), 496–513. 10.1016/j.ccell.2020.03.009 32289273 PMC7169993

[B13] Hortobagyi G. N. Stemmer S. M. Burris H. A. Yap Y. S. Sonke G. S. Hart L. (2022). Overall survival with ribociclib plus letrozole in advanced breast cancer. N. Engl. J. Med. 386 (10), 942–950. 10.1056/NEJMoa2114663 35263519

[B14] Hua M. Xiong F. Chong S. Zhang Z. Liu Q. Hou J. (2024). Abemaciclib increases the risk of venous thromboembolism in breast cancer: integrate meta-analysis, pharmacovigilance database analysis, and *in vitro* validation. Cancer Treat. Rev. 130, 102827. 10.1016/j.ctrv.2024.102827 39278067

[B15] Khorana A. A. Francis C. W. Culakova E. Kuderer N. M. Lyman G. H. (2007). Thromboembolism is a leading cause of death in cancer patients receiving outpatient chemotherapy. J. Thromb. Haemost. 5 (3), 632–634. 10.1111/j.1538-7836.2007.02374.x 17319909

[B16] Kinoshita S. Hosomi K. Yokoyama S. Takada M. (2020). Time-to-onset analysis of amiodarone-associated thyroid dysfunction. J. Clin. Pharm. Ther. 45 (1), 65–71. 10.1111/jcpt.13024 31400296

[B17] Liu W. Ye X. Shan H. Wang M. Wang Y. Guo Z. (2024). Unraveling the spectrum of ocular toxicity with oxaliplatin: clinical feature analysis of cases and pharmacovigilance assessment of the US Food and drug administration adverse event reporting system database. Clin. Ther. 46 (12), 1049–1058. 10.1016/j.clinthera.2024.09.019 39428274

[B18] Liu Y. S. Dong K. Park C. (2023). Risk of cardiovascular events with cyclin-dependent kinases 4 and 6 (CDK 4/6) inhibitors among patients with advanced breast cancer: a systematic review and network meta-analysis. Rev. Cardiovasc Med. 24 (11), 309. 10.31083/j.rcm2411309 39076428 PMC11262445

[B19] Matutino A. Joy A. A. Brezden-Masley C. Chia S. Verma S. (2018). Hormone receptor-positive, HER2-negative metastatic breast cancer: redrawing the lines. Curr. Oncol. 25 (Suppl. 1), S131-S141–S141. 10.3747/co.25.4000 29910656 PMC6001771

[B20] Morrison L. Loibl S. Turner N. C. (2024). The CDK4/6 inhibitor revolution - a game-changing era for breast cancer treatment. Nat. Rev. Clin. Oncol. 21 (2), 89–105. 10.1038/s41571-023-00840-4 38082107

[B21] Murad B. Reis P. C. A. Deberaldini Marinho A. Marin Comini A. C. Pinheiro Xavier D. Mella Soares Pessoa B. (2024). QTc prolongation across CDK4/6 inhibitors: a systematic review and meta-analysis of randomized controlled trials. JNCI Cancer Spectr. 8 (5), pkae078. 10.1093/jncics/pkae078 39254653 PMC11460542

[B22] Onesti C. E. Jerusalem G. (2021). CDK4/6 inhibitors in breast cancer: differences in toxicity profiles and impact on agent choice. A systematic review and meta-analysis. Expert Rev. Anticancer Ther. 21 (3), 283–298. 10.1080/14737140.2021.1852934 33233970

[B23] Oyakawa T. Inagaki L. Hua Z. Ebihara A. Takano T. Ohno S. (2021). Myocardial dysfunction caused by abemaciclib: a case report. Int. Cancer Conf. J. 10 (4), 324–328. 10.1007/s13691-021-00500-3 34567946 PMC8421487

[B24] Papageorgiou C. Zagouri F. Tampakis K. Georgakopoulou R. Manios E. Kafouris P. (2021). Vascular inflammation and cardiovascular burden in metastatic breast cancer female patients receiving hormonal treatment and CDK 4/6 inhibitors or everolimus. Front. Cardiovasc Med. 8, 638895. 10.3389/fcvm.2021.638895 33732735 PMC7959765

[B25] Prat A. Solovieff N. André F. O'Shaughnessy J. Cameron D. A. Janni W. (2024). Intrinsic subtype and overall survival of patients with advanced HR+/HER2- breast cancer treated with ribociclib and ET: correlative analysis of MONALEESA-2, -3, -7. Clin. Cancer Res. 30 (4), 793–802. 10.1158/1078-0432.CCR-23-0561 37939142 PMC10870119

[B26] Raschi E. Fusaroli M. Ardizzoni A. Poluzzi E. De Ponti F. (2021). Thromboembolic events with cyclin-dependent kinase 4/6 inhibitors in the FDA adverse event reporting system. Cancers (Basel) 13 (8), 1758. 10.3390/cancers13081758 33917020 PMC8067683

[B27] Rhee J. W. Ky B. Armenian S. H. Yancy C. W. Wu J. C. (2020). Primer on biomarker discovery in cardio-oncology: application of omics technologies. JACC CardioOncol 2 (3), 379–384. 10.1016/j.jaccao.2020.07.006 33073248 PMC7560982

[B28] Rugo H. S. Huober J. García-Sáenz J. A. Masuda N. Sohn J. H. Andre V. A. M. (2021). Management of abemaciclib-associated adverse events in patients with hormone receptor-positive, human epidermal growth factor receptor 2-negative advanced breast cancer: safety analysis of MONARCH 2 and MONARCH 3. Oncologist 26 (1), e53–e65. 10.1002/onco.13531 32955138 PMC7794176

[B29] Santoni M. Occhipinti G. Romagnoli E. Miccini F. Scoccia L. Giulietti M. (2019). Different cardiotoxicity of palbociclib and ribociclib in breast cancer: gene expression and pharmacological data analyses, biological basis, and therapeutic implications. BioDrugs 33 (6), 613–620. 10.1007/s40259-019-00382-1 31529317

[B30] Siegel R. L. Miller K. D. Wagle N. S. Jemal A. (2023). Cancer statistics, 2023. CA Cancer J. Clin. 73 (1), 17–48. 10.3322/caac.21763 36633525

[B31] Slamon D. Lipatov O. Nowecki Z. McAndrew N. Kukielka-Budny B. Stroyakovskiy D. (2024b). Ribociclib plus endocrine therapy in early breast cancer. N. Engl. J. Med. 390 (12), 1080–1091. 10.1056/NEJMoa2305488 38507751

[B32] Slamon D. J. Diéras V. Rugo H. S. Harbeck N. Im S. A. Gelmon K. A. (2024a). Overall survival with palbociclib plus letrozole in advanced breast cancer. J. Clin. Oncol. 42 (9), 994–1000. 10.1200/JCO.23.00137 38252901 PMC10950136

[B33] Waks A. G. Winer E. P. (2019). Breast cancer treatment: a review. JAMA 321 (3), 288–300. 10.1001/jama.2018.19323 30667505

[B34] Watson N. W. Shatzel J. J. Al-Samkari H. (2023). Cyclin-dependent kinase 4/6 inhibitor-associated thromboembolism: a critical evaluation of the current evidence. J. Thromb. Haemost. 21 (4), 758–770. 10.1016/j.jtha.2022.12.001 36696184 PMC10065951

[B35] Watson N. W. Wander S. A. Shatzel J. J. Al-Samkari H. (2022). Venous and arterial thrombosis associated with abemaciclib therapy for metastatic breast cancer. Cancer 128 (17), 3224–3232. 10.1002/cncr.34367 35767226 PMC10042227

[B36] West M. T. Smith C. E. Kaempf A. Kohs T. C. L. Amirsoltani R. Ribkoff J. (2021). CDK 4/6 inhibitors are associated with a high incidence of thrombotic events in women with breast cancer in real-world practice. Eur. J. Haematol. 106 (5), 634–642. 10.1111/ejh.13590 33527479 PMC8087188

[B37] Wilcox N. S. Amit U. Reibel J. B. Berlin E. Howell K. Ky B. (2024). Cardiovascular disease and cancer: shared risk factors and mechanisms. Nat. Rev. Cardiol. 21 (9), 617–631. 10.1038/s41569-024-01017-x 38600368 PMC11324377

[B38] Yan Y. Wu B. Wang L. (2024). A real-world pharmacovigilance study of QT interval prolongation and Torsades de Pointes associated with CDK4/6 inhibitors in breast cancer patients: findings from the FDA adverse event reporting system. Expert Opin. Drug Saf. 23 (9), 1191–1198. 10.1080/14740338.2024.2307375 38482864

[B39] Yang C. Zhao W. Chen H. Yao Y. Zhang J. (2024). Cardiac adverse events associated with lacosamide: a disproportionality analysis of the FAERS database. Sci. Rep. 14 (1), 16202. 10.1038/s41598-024-67209-0 39003359 PMC11246456

[B40] Yang L. Xue J. Yang Z. Wang M. Yang P. Dong Y. (2021). Side effects of CDK4/6 inhibitors in the treatment of HR+/HER2-advanced breast cancer: a systematic review and meta-analysis of randomized controlled trials. Ann. Palliat. Med. 10 (5), 5590–5599. 10.21037/apm-21-1096 34107710

[B41] Zhang C. Shen G. Li S. Ma F. Li H. Tang Y. (2025). Cardiovascular events associated with CDK4/6 inhibitors: a safety meta-analysis of randomized controlled trials and a pharmacovigilance study of the FAERS database. Am. J. Cardiovasc Drugs 25 (3), 373–388. 10.1007/s40256-024-00709-6 39695060

